# Changes in the Expression of TBP-2 in Response to Histone Deacetylase Inhibitor Treatment in Human Endometrial Cells

**DOI:** 10.3390/ijms22031427

**Published:** 2021-01-31

**Authors:** Hye In Kim, Seok Kyo Seo, Seung Joo Chon, Ga Hee Kim, Inha Lee, Bo Hyon Yun

**Affiliations:** 1 Department of Obstetrics and Gynecology, Severance Hospital, Yonsei University College of Medicine, Seoul 03722, Korea; HYEIN24@yuhs.ac (H.I.K.); TUDEOLSEO@yuhs.ac (S.K.S.); IHLEE86@yuhs.ac (I.L.); 2 Institute of Women’s Life Medical Science, Yonsei University College of Medicine, Seoul 03722, Korea; SUWOAL@yuhs.ac; 3 Department of Obstetrics and Gynecology, Gil Hospital, Gachon University College of Medicine, Inchon 21565, Korea; sjchon@gilhospital.com

**Keywords:** apoptosis, endometriosis, oxidative stress, suberoylanilide hydroxamic acid, thioredoxin

## Abstract

Histone deacetylase inhibitors (HDACi) induce apoptosis preferentially in cancer cells by caspase pathway activation and reactive oxygen species (ROS) accumulation. Suberoylanilide hydroxamic acid (SAHA), a HDACi, increases apoptosis via altering intracellular oxidative stress through thioredoxin (TRX) and TRX binding protein-2 (TBP-2). Because ROS accumulation, as well as the redox status determined by TBP-2 and TRX, are suggested as possible mechanisms for endometriosis, we queried whether SAHA induces apoptosis of human endometrial cells via the TRX–TBP-2 system in endometriosis. Eutopic endometrium from participants without endometriosis, and ectopic endometrium from patients with endometriosis, was obtained surgically. Human endometrial stromal cells (HESCs) and Ishikawa cells were treated with SAHA and cell proliferation was assessed using the CCK-8 assay. Real-time PCR and Western blotting were used to quantify TRX and TBP-2 mRNA and protein expression. After inducing oxidative stress, SAHA was applied. Short-interfering TRX (SiTRX) transfection was performed to see the changes after TRX inhibition. The mRNA and protein expression of TBP-2 was increased with SAHA concentrations in HESCs significantly. The mRNA TBP-2 expression was decreased after oxidative stress, upregulated by adding 2.5 μM of SAHA. The TRX/TBP-2 ratio decreased, apoptosis increased significantly, and SiTRX transfection decreased with SAHA. In conclusion, SAHA induces apoptosis by modulating the TRX/TBP-2 system, suggesting its potential as a therapeutic agent for endometriosis.

## 1. Introduction

Endometriosis is a gynecological disease that affects women of reproductive age. Its incidence varies from 2 to 52%, differing among the following groups: a fertile control group showed a prevalence of about 6% in the 1990s, a chronic pelvic pain group about 15%, and an infertile group about 21% [1]. Recently, the prevalence of endometriosis was reported to be 25 to 50% in infertile women and 71 to 87% in women with chronic pelvic pain [2,3]. According to the global data, the prevalence of endometriosis increased by 6.4% from 1998 to 2013 [4]. The increase in laparoscopic surgery interventions may have increased the diagnosis of endometriosis. However, considering that endometriosis diagnosis is delayed by about 7 to 10 years from initial symptom development and the prevalence is underestimated, the increase in the incidence rate may be a global trend [5,6].

The pathophysiology of endometriosis is not yet fully understood. There are three leading theories to explain how endometriosis develops. Retrograde menstruation theory, also known as Sampson’s theory, suggests that the endometrial cell retrogresses through salpinx and implants into the peritoneal cavity [7]. Coelomic metaplasia theory proposes that endometriosis arises due to the metaplasia of mesothelial cells on the peritoneum into endometrial cells [8]. Embryonic origin theory hypothesizes that the endometrial cells in the peritoneal cavity were present from the development, and they spread with exposure to hormones, oxidative stress or other environmental factors [9].

The management of endometriosis at present is not very different from that 30 years ago. Surgery is the only effective treatment option, which increases fertility and effectively assists in pelvic pain management. Although medical treatment is emphasized more than ever in terms of controlling pelvic pain, preserving fertility, and preventing recurrence, there are no commercialized drugs available other than hormonal treatment. Individualized treatment for endometriosis is a critical need as research keeps revealing the various mechanisms, subtypes, and prognoses of endometriosis. Patients who seek to conceive or suffer from the side effects of hormonal treatment need other options; however, currently, these are very few.

Histone deacetylase inhibitors (HDACis) were developed as targeted anticancer agents that selectively affect cancer cells [10]. Suberoylanilide hydroxamic acid (SAHA) is one of the most studied HDACis; it exerts significant anticancer activity in hematologic malignancies, breast cancer and lung cancer, and is well tolerated by patients, as shown in clinical trials [11,12,13,14]. For ovarian cancer, HDACi showed effectiveness in combination with other cancer drugs, such as DNA methyltransferases (DNMTs). The combination overcame the platinum resistance and showed an improved response to immune checkpoint therapy in a mouse model [15,16,17]. Histone acetylation is a crucial process in gene transcription, and the regulation of enzymes that modulate histone acetylation explains the overgrowth of cells in various tumors. HDACis induce apoptosis preferentially in cancer cells, possibly via upregulating pro-death genes or downregulating pro-survival genes. In breast and ovarian cancer, HDACis showed to increase the effectiveness of cisplatin through a BRCA1-dependent mechanism, which is being studied in various clinical trials for new target therapies [18]. Moreover, HDACis increase cell death by increasing reactive oxygen species (ROS) [19], resulting from the regulation of the redox status by thioredoxin-binding protein (TBP)-2 and thioredoxin (TRX) [20]. The redox status and oxidative stress were previously reported as potential mechanisms of endometriosis by our group [21], which suggested the aberrant expression of TRX and TBP-2 in endometrial stromal cells from women with endometriosis.

Non-hormonal options, such as modulators, which affect angiogenesis, inflammatory pathways, fibrosis, and oxidative stress, and immune-modulators have been suggested as novel drugs for endometriosis treatment. The hypothesis suggesting that the immunological mechanism involved in the development of endometriosis may initiate early development, and that hormones contribute to the inflammatory response, is gaining momentum [22]. Previously, we proposed that oxidative stress may induce the proliferation of eutopic endometrial stromal cells, suppress apoptosis, and activate innate immunity to initiate inflammation [23]. In the current study, we focused on HDACis as a potential modulator to increase apoptosis in endometriotic cells and alter endometrial stromal cells. We also aimed to examine whether SAHA induces apoptosis specifically in endometrial stromal cells by changing the redox status via the regulation of TRX and TBP-2. We collected eutopic and ectopic endometrium. After cell culture, we performed the CCK-8 assay for cell proliferation, flow cytometry for apoptosis, real-time PCR and western blotting for the quantification of TRX and TBP-2, and short-interfering RNA transfection for TRX/TBP-2 ratio and apoptosis.

## 2. Results

### 2.1. TRX and TBP-2 Changes to SAHA Treatment in HESCs and Ishikawa Cells

**Figure 1:**
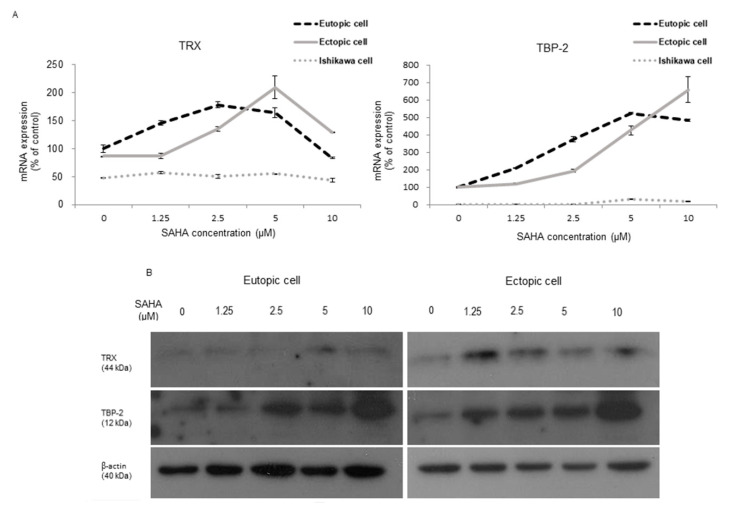
TRX and TBP-2 expression in endometrial cells, before and after treating with SAHA. (**A**) Relative mRNA levels of TRX, TBP-2 was presented according to the treatment doses. There was no difference in the mRNA level in Ishikawa cells, but there was a significant increase in HESCs up to 5 μM of SAHA treatment. (**B**) Representative immunoblots revealed increased TRX and TBP-2 in eutopic and ectopic HESCs, by Western blot; β-actin was used as an internal control.

SAHA was administered to eutopic and ectopic human endometrial stromal cells (HESCs) and Ishkawa cells, to examine TRX and TBP expression in mRNA and protein level in naïve cells. The mRNA expression of TRX was increased in the stromal cells but not in the Ishikawa cells (Figure 1A). The upregulation of TBP-2 mRNA and protein synthesis was significant in HESCs, but not in Isihikawa cells (Figure 1B).

### 2.2. Changes after SAHA Treatment in the Oxidative Stress-Induced Altered Endometrial Stromal and Epithelial Cells

**Figure 2:**
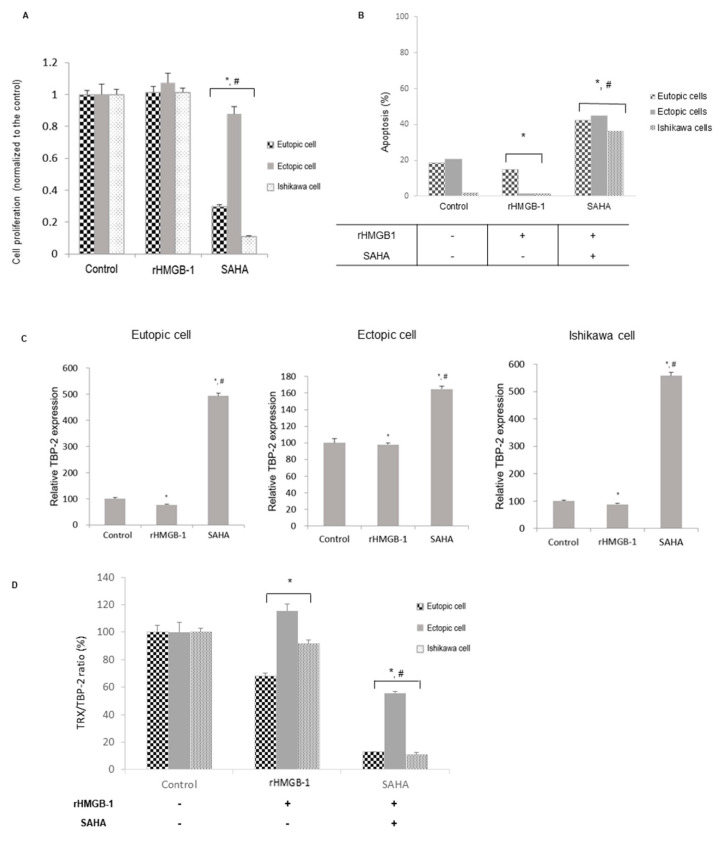
Endometrial cell proliferation and TRX, TBP-2 expression after SAHA treatment in stress-induced cells. Oxidative stress was applied using rHMGB-1 10 ng/mL for 24 h. (**A**) Endometrial cell proliferation was measured after 48 h of treatment with 2.5 μM SAHA, which revealed slight increases in three types of cells. (**B**) The proportion of apoptotic cells was measured using flow cytometry. Apoptosis was decreased after treating with rHMGB-1, but was soon overwhelmed with SAHA treatment. (**C**) The relative mRNA expression levels of TBP-2 showed significant reductions after rHMGB-1 treatment compared to the controls. SAHA increased TBP-2 mRNA expression significantly. (**D**) The TRX/TBP-2 ratio was increased in ectopic HESCs after rHMGB-1 treatment only, and then significantly decreased after SAHA treatment. *, *p* < 0.05 compared to the control; #, *p* < 0.05 compared to the rHMGB-1-treated cells.

After oxidative stress was induced using rHMGB-1, cell proliferation was increased in three groups (Figure 2A). Concurrently, oxidative stress led to decreased apoptosis significantly in three groups, especially in the ectopic HESCs (Figure 2B, Figure S1). However, SAHA treatment reversed this effect; moreover, higher levels of apoptosis were observed in the SAHA-treated groups than in the controls. The mRNA expression of TBP-2 showed a significant decrease after rHMGB-1 treatment in the stromal or epithelial cells (Figure 2C). However, after SAHA treatment, the mRNA expression of TBP-2 increased significantly in the three groups. The TRX/TBP-2 ratio was increased in the ectopic HESCs after rHMGB-1 treatment; however, soon after SAHA treatment, the TRX/TBP-2 ratio showed a significant decrease (Figure 2D).

### 2.3. SAHA Treatment Inhibits TRX Gene Expression in the Altered Endometrial Cells

**Figure 3:**
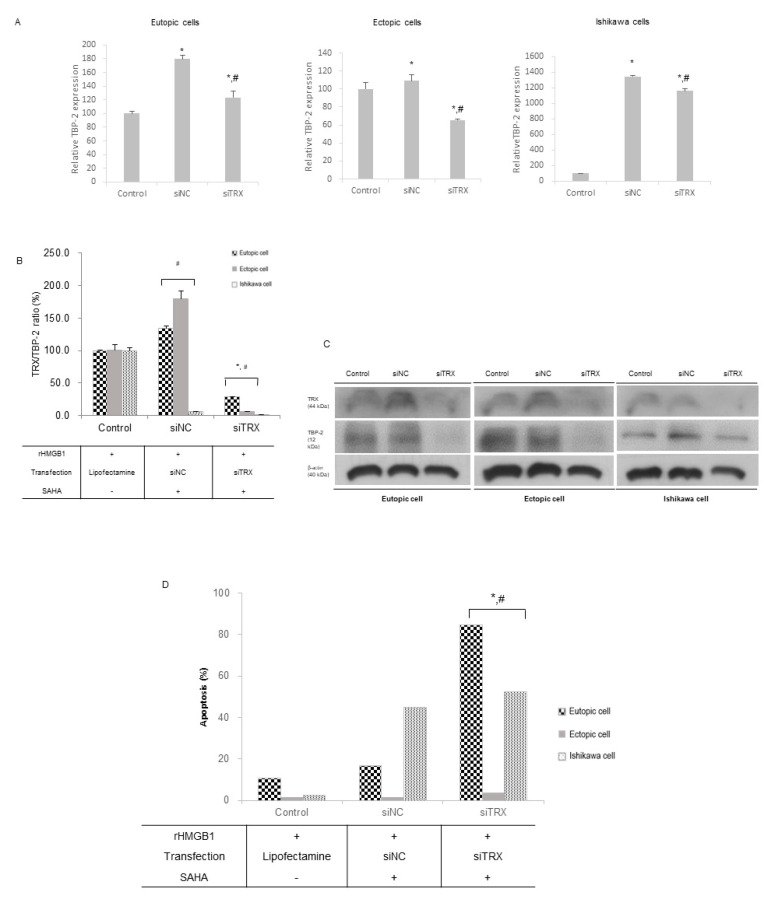
Changes after oxidative stress in siTRX transfected endometrial cells. The cells were treated with 10 mg/mL of rHMGB-1 for 24 h, followed by 2.5 μM SAHA treatment for 48 h. (**A**) The mRNA expression of TBP-2 was modified after siTRX transfection. (**B**) The TRX/TBP-2 ratio of mRNA expression revealed a significant decrease in siTRX-transfected cells. (**C**) Western blot was concordant with RT-PCR result. (**D**) Apoptosis was significantly increased in siTRX -transfected cell treated with SAHA, compared to non-SAHA-treated cells (control), and siNC (negative control) cells, respectively. *, *p* < 0.05 compared to the control; #, *p* < 0.05 compared to siNC.

Eutopic and ectopic HESCs, along with Ishikawa cells, were transfected with siRNA to inhibit TRX gene expression. TRX mRNA expression was inhibited by over 50% in the three cell groups (Figure S2). Along with the control cells, the SAHA-treated cells were transfected with siNC and siTRX mRNA. Cells with inhibited TRX expression showed significantly lower TBP-2 expression levels at the mRNA and protein levels compared to those of siNC after SAHA treatment (Figure 3A,C). The TRX/TBP-2 ratio was increased after SAHA treatment in the stroma cells but not in the Ishikawa cells (Figure 3B). After the suppression of the mRNA expression of TRX, the TRX/TBP-2 ratio decreased significantly. The levels of apoptosis were significantly higher in the eutopic and ectopic HESCs, as well as in the Ishikawa cells (Figure 3D) with siTRX transfection after SAHA treatment, than in those with siNC transfection.

## 3. Discussion

Endometriosis is one of the diseases for which early diagnosis and treatment are important because of their complications, such as symptoms and sequelae, if untreated. Even though it has been studied for more than 160 years, there are still controversies and questions regarding its diagnosis, pathogenesis, treatment, and prognosis [24]. Surgery is still the only treatment which improves fertility and reduces chronic pelvic pain. Recently, the medical treatment recommends preserving the ovarian reserve, but hormonal treatment, of which many people have a negative view, is the only option. Therefore, a personalized treatment option for endometriosis is in need.

SAHA is one type of HDACis which is used as a cancer drug for hematologic malignancies, breast cancer, lung cancer, and ovarian cancer [11,12,13,14]. HDACi induces apoptosis in cancer cells by increasing ROS and regulating the redox status by TBP-2 and TRX, which was also suggested as a potential pathophysiology of endometriosis [21]. It is assumed that SAHA can also play a role as a treatment option for endometriosis.

In the present study, we observed that SAHA enhanced HESC apoptosis by changing TBP-2 expression. TBP-2 modulates the intracellular TRX–oxidation system, thus counteracting the oxidative stress induced by TRX binding [25]. TRX is a ribonucleotide reductase that acts as a scavenger of ROS, providing hydrogen molecules to many protein targets [26]. Localized decreased apoptosis in the ectopic endometrium is a unique characteristic of endometriosis [27]. In previous studies on cancer cells, SAHA suppressed cell proliferation in multiple myeloma cells (which the authors present as a ”transformed” cell), normal breast fibroblasts, and lung fibroblast cells [28]. The mechanism underlying the apoptosis mediated by SAHA was explained by various theories, including increased intracellular TRX accumulation and upregulated TBP-2 expression [19].

The intracellular TRX–oxidation system modulates the oxidation state of cells so that the cells may survive or die. TBP-2 is a binding protein of TRX; it is a negative regulator that inhibits the reducing activity of TRX. In endometriosis, the decrease in TBP-2 expression was greater in eutopic HESCs from endometriosis patients than in the controls [21]. Although there were minimal changes in TRX expression, the TRX/TBP-2 ratio was significantly higher in the endometriosis group than in the controls. Considering the relationship between TRX and TBP-2, the TRX/TBP-2 ratio may reflect the functional activity of TRX. In our study, SAHA induced a significant increase in the mRNA and protein expression of TBP-2 in the stromal cells (eutopic and ectopic HESCs) and minimal changes in the epithelial cells (Ishikawa cells). In the eutopic and ectopic HESCs, TRX expression was increased after SAHA treatment, which was different from that observed in the Ishikawa cells. This finding agrees with that of a previous report, which showed that TRX accumulation increases after SAHA treatment in normal cells [28].

After oxidative stress was induced in the cells, the cell viability increased in the three groups. TBP-2 expression was previously shown to decrease after rHMGB-1 treatment, just like TBP-2 expression, in eutopic HESCs from patients with endometriosis [21]. In our study, after SAHA treatment, we observed a significant decrease in cell viability in the three groups, along with an increase in apoptosis. The mRNA and protein expression of TBP-2 was increased markedly. The relative TRX activity, shown by the TRX/TBP-2 ratio, increased after oxidative stress induction, especially in the ectopic HESCs; however, the TRX/TBP-2 ratio was significantly lower than that before treatment in the three groups.

The advantage that SAHA possesses over other drugs is its cell-specific activity, according to the cellular redox state. SAHA induces cell apoptosis mainly in transformed cells, minimally affecting normal cells. Lately, multiple clinical studies regarding the application of SAHA in various cancer therapies have been conducted [11,29,30]. In cancer cells, when the TRX gene is suppressed, sensitivity to SAHA increases. Reduced TRX is probably caused by the downregulation of TBP-2 expression [19]. It is suggested that decreased TRX may not protect the cell from oxidative stress; thus, cell death increases owing to the altered TRX-oxidation system. Our results also revealed significantly higher levels of apoptosis after siTRX transfection than after siNC transfection in the three groups. Contrary to previous reports, apoptosis was increased not only in the Ishikawa cells and ectopic HESCs, but also in the eutopic HESCs. The finding may be explained by the characteristics of the endometrial cell itself. Unlike other organ, endometrial cells proliferate, and drop out rapidly and actively every month according to the menstrual cycle. Therefore, there is a fast turnover of these cells—proliferation, apoptosis and withdrawal—in nature.

Although TBP-2 and TRX expression in the three groups after rHMGB-1 and SAHA treatment did not show a sequential change in the eutopic and ectopic HESCs and Ishikawa cells, a consistent TRX/TBP-2 ratio was shown. After SAHA treatment in the oxidative stress-induced cells, the TRX/TBP-2 ratio decreased significantly, and apoptosis increased concordantly. When TRX expression was suppressed, apoptosis following SAHA treatment increased significantly. The findings suggest the possibility of more severe cell alteration via oxidative stress and increased apoptosis induced by SAHA. The discrepancy may be attributed to the manner in which SAHA works in cells, involving not only the TRX–oxidative system but also epigenetic changes. In the ectopic endometrial stromal cells, SAHA downregulates the G-protein-coupled estrogen receptor [31].

One limitation of our study is that the design was limited to in vitro experiments. Although the cell culture and molecular examinations may not reflect the characteristics of endometriosis thoroughly, stromal cells have been used to study its pathophysiology for years [32,33]. Except for those ones who desire pregnancy, all the patients who provided the endometriosis samples were given postoperative adjuvant medical treatment: GnRH agonist or progestin after their diagnosis. However, their long-term follow-up data were not compiled for research purposes.

### Conclusions

Our study suggests SAHA as a potential novel drug for endometriosis treatment, as it has been used in anticancer therapy trials. Although our study did not successfully show the different cell responses to SAHA among the eutopic and ectopic HESCs and Ishikawa cells, the increased levels of apoptosis suggest its significant potential.

## 4. Materials and Methods

**Figure 4:**
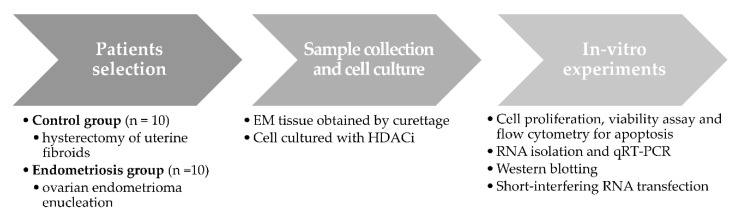
The workflow diagram.

The overall study workflow is summarized in Figure 4. To examine the manner in which SAHA affects cell apoptosis selectively, eutopic endometrial stromal cells, ectopic endometrial stromal cells, and endometrial epithelial cells (Ishikawa cell line) were selected. Cell proliferation was assessed using a CCK-8 proliferation assay kit, and cell viability was assessed using the MTT assay. The apoptosis of human endometrial stromal cells (HESCs) was assessed using flow cytometry. Quantitative real-time polymerase chain reaction (qRT-PCR) and Western blot analyses were used to quantify the mRNA and protein expression levels of TRX and TBP-2. After inducing oxidative stress by adding rHMGB-1 to the cells, SAHA treatment was applied, and the subsequent changes in cell proliferation and viability as well as TRX and TBP-2 expression were examined. After inhibiting TRX via siRNA transfection, the cell proliferation and apoptosis induced by SAHA were compared.

### 4.1. Participants

For cell culture, eutopic endometrium specimens were obtained from 10 patients without endometriosis following a hysterectomy of uterine fibroids, and ectopic endometrium cells were obtained from 10 patients who had undergone ovarian endometrioma enucleation. All participants were of reproductive age and reportedly had regular menstrual cycles every 28–30 days. None of the participants received any hormonal treatment for at least 3 months before surgery. The study was approved by the institutional review board of Severance Hospital, Yonsei University College of Medicine (IRB No. 4-2015-0466; Approval date: 14 July 2015). All participants who provided endometrial tissue for cell culture provided written informed consent.

### 4.2. Sample Collection and Cell Culture

Endometrial tissue was obtained by curettage from all participants at the beginning of the hysterectomy process in the operation room. Tissue (1 × 1 cm) from the endometrioma was excised right after enucleation. The specimens were transferred into a buffered saline solution directly after isolation and immediately sent to the laboratory for analysis. HESCs and human endometrial epithelial cells (Ishikawa cell line, Sigma-Aldrich, St. Louis, MO, USA) were cultured using the same method, which was previously described [23]. Ishikawa cells (1 × 10^6^) and HESCs (5 × 10^4^) were grown in Dulbecco’s modified Eagle’s medium/F12 (DMEM/F12, HyClone, Logan, UT, USA), treated with 10 ng/mL of rHMGB-1 diluted in 2 mL of culture medium, and incubated at 37 °C in a 5% CO_2_ incubator for 24 h. The HDACi was added at different concentrations 24 h after seeding, and the cells were cultured for 48 h. The cells were recovered after trypsinization and washed; then, cell proliferation and viability were determined.

### 4.3. Drugs and Chemicals

SAHA (Cayman, Ann Arbor, MI, USA) was chosen as the HDACi, and rHMGB-1 (Sino, Beijing, China) (10 ng/mL) was used to induce oxidative stress. After determining the dosage, 2.5 μM SAHA was added to the culture of stressed cells.

### 4.4. Cell Proliferation and Viability, Analysis of Apoptosis

Endometrial cells were seeded in 6-well tissue culture plates at a density of 1 × 10^5^ cells/well. The culture medium was changed to DMEM/F12 containing 2% fetal bovine serum (FBS; Gibco, Invitrogen) after 24 h of incubation. After changing the media, endometrial cells were treated with SAHA at concentrations of 1.25, 2.5, 5, and 10 µM for 48 h. Cells were treated with rHMGB-1 (10 ng/mL) for 24 h; subsequently, 2.5 µM SAHA was added for 48 h to compare the differences after inducing oxidative stress. Subsequently, 100 μL of CCK-8 (Cell Counting Kit-8; Dojindo, Japan) was added to each well, and the plates were incubated at 37 °C for 1 h. Supernatants were transferred to 96-well plates after incubation, and the OD was measured at 450 nm using a VersaMax microplate reader (Molecular Devices, Sunnyvale, CA, USA) to calculate the cell proliferation rates. To measure cell viability, we added 100 μL of MTT solution (Sigma, St Louis, MO, USA) to each well, and the culture plates were incubated at 37 °C for 4 h in a 5% CO_2_ incubator. Supernatants were harvested for subsequent Western blot analysis. After removing the medium, 500 μL of dimethyl sulfoxide (DMSO; Sigma) was added to each well, and the plates were then incubated for 10 min on a shaker. Finally, the OD was measured at 562 nm using a VersaMax microplate reader. HESCs (2 × 10^5^) and Ishikawa cells (5 × 10^5^) were seeded in 100 mm culture dishes and incubated for 48 h in DMEM/F12 containing 10% FBS. The medium was replaced with DMEM/F12 containing 2% FBS, and the cells were treated with rHMGB-1 and SAHA. The cells were then stained with 5 mL fluorescein isothiocyanate/annexin V and propidium iodide (PI) according to the manufacturer’s protocol. Apoptotic cells were identified using flow cytometry (FC 500; Beckman Coulter, Fullerton, CA, USA), and the data were analyzed using WinMDI v.2.9 software (The Scripps Research Institute, San Diego, CA, USA).

### 4.5. RNA Isolation and qRT-PCR

Cell-free total RNA was extracted from cell lysates using an RNeasy mini kit (Qiagen, Hilden, Germany). cDNA was synthesized from 1 μg total RNA using oligo-dT primers (Invitrogen). qRT-PCR of TRX and TBP-2 was carried out using ABI StepOnePlus (Applied Biosystems, Foster City, CA, USA) and SYBR Green RT-PCR Master mix (Toyobo, Osaka, Japan). The PCR conditions were as follows: 95 °C for 10 min; 40 cycles of 95 °C for 15 s, 60 °C for 1 min, and 95 °C for 15 s. The primers used for TRX, TBP-2, and glyceraldehyde 3 phosphate dehydrogenase (GAPDH) were as follows: TRX, 5′-GGT GTG GGC CTT GCA AAA T-3′ (sense) and 5′-AAT ATC ACG TTG GAA TAC TTT TCA GAG A-3′ (antisense); TBP-2, 5′-GGG TGA TAG TGG AGG TGT GTG AA-3′ (sense) and 5′-CCG CAA GCC AGG ATC CTA A-3′ (antisense); and GAPDH, 5′-TCG ACA GTC AGC CGC ATC TTC TTT-3′ (sense) and 5′-ACC AAA TCC GTT GAC TCC GAC CTT-3′ (antisense). Data were normalized to the expression of GAPDH, and the PCR products were separated on a 1.5% agarose gel containing ethidium bromide. The bands were quantified by densitometry using ImageJ software (National Institutes of Health, USA).

### 4.6. Western Blotting

Cells were lysed using a radioimmunoprecipitation assay buffer (RIPA buffer; Intron, iNtRON Biotechnology, Sungnam, Korea) containing a protease inhibitor cocktail (Cell Signaling Technology, Beverly, MA, USA), mixed, and divided into 40 μL aliquots. The lysates were collected and centrifuged at 13,000 rpm at 4 °C for 30 min, and the amount of protein in the supernatants was quantified using a BCA protein assay kit (Thermo Scientific, Hudson, NH, USA). Western blot analysis was then performed using the harvested supernatants. Thirty micrograms of each lysate were boiled in 5× buffer; the supernatants were then separated using sodium dodecyl sulfate–polyacrylamide gel electrophoresis (SDS-PAGE) (8%) and transferred to polyvinylidene fluoride (PVDF) membranes (Millipore, Eschborn, Germany). After blocking with BSA at room temperature for 1 h, the membranes were incubated with specific primary antibodies against TRX (monoclonal anti-rabbit, 1:50,000; Abcam, Cambridge, MA, USA), TBP-2 (polyclonal anti-rabbit, 0.3 μg/mL, GeneTex, Irvine, CA, USA), and GAPDH (monoclonal anti-mouse, 0.1 μg/mL, Millipore) overnight at 4 °C. The membranes were then incubated with anti-mouse antibodies (IgG, 0.27 μg/mL, Jackson, West Grove, PA, USA) and anti-rabbit antibodies (IgG, 0.27 μg/mL, Jackson) conjugated with horseradish peroxidase (HRP) for 1 h at room temperature. Detection was facilitated by the use of an enhanced chemiluminescence (ECL) solution (Advansta, San Francisco, CA, USA), and the bands were quantified by densitometry using ImageJ software.

### 4.7. Short-Interfering RNA Transfection

TRX short-interfering RNA (siRNA) was purchased from Bioneer Dharmacon (Lafayette, Colorado, CO, USA). HESCs were transfected with siRNA oligonucleotides using Lipofectamine RNAiMAX (Gibco, Invitrogen, Carlsbad, CA, USA) according to the manufacturer’s recommendations. After 4 h, the cells were washed and incubated overnight in complete medium with 10 ng/mL rHMGB-1. On the following day, the cells were treated with 2.5 μM SAHA or vehicle for 48 h. The effect of TRX downregulation was evaluated using RT-PCR and Western blot analysis; coordinating cell proliferation was assessed.

### 4.8. Statistical Analysis

All experiments were performed in triplicate. Kruskal–Wallis tests with Dunn’s procedure for multiple comparisons were performed to determine the significance of the differences between groups. Statistical analyses were performed using SPSS 22.0 (IBM, Armonk, NY, USA). *p* values less than 0.05 were considered statistically significant.

## Data Availability

The data presented in this study are openly available in FigShare at [10.6084/m9.figshare.13669802.v2].

## Supplementary Materials

The following are available online at https://www.mdpi.com/1422-0067/22/3/1427/s1, Figure S1: Endometrial cell proliferation; Figure S2: Eutopic and ectopic HESCs, along with Ishikawa cells, transfected with siRNA to inhibit TRX gene expression; Figure S3: Endometrial cell proliferation.
