# Supplementary figures and images for: Changes in the Expression of TBP-2 in Response to Histone Deacetylase Inhibitor Treatment in Human Endometrial Cells
Source: Int J Mol Sci. 2021 Jan 31;22(3):1427. doi: 10.3390/ijms22031427 (PMC7866992; doi:10.3390/ijms22031427)

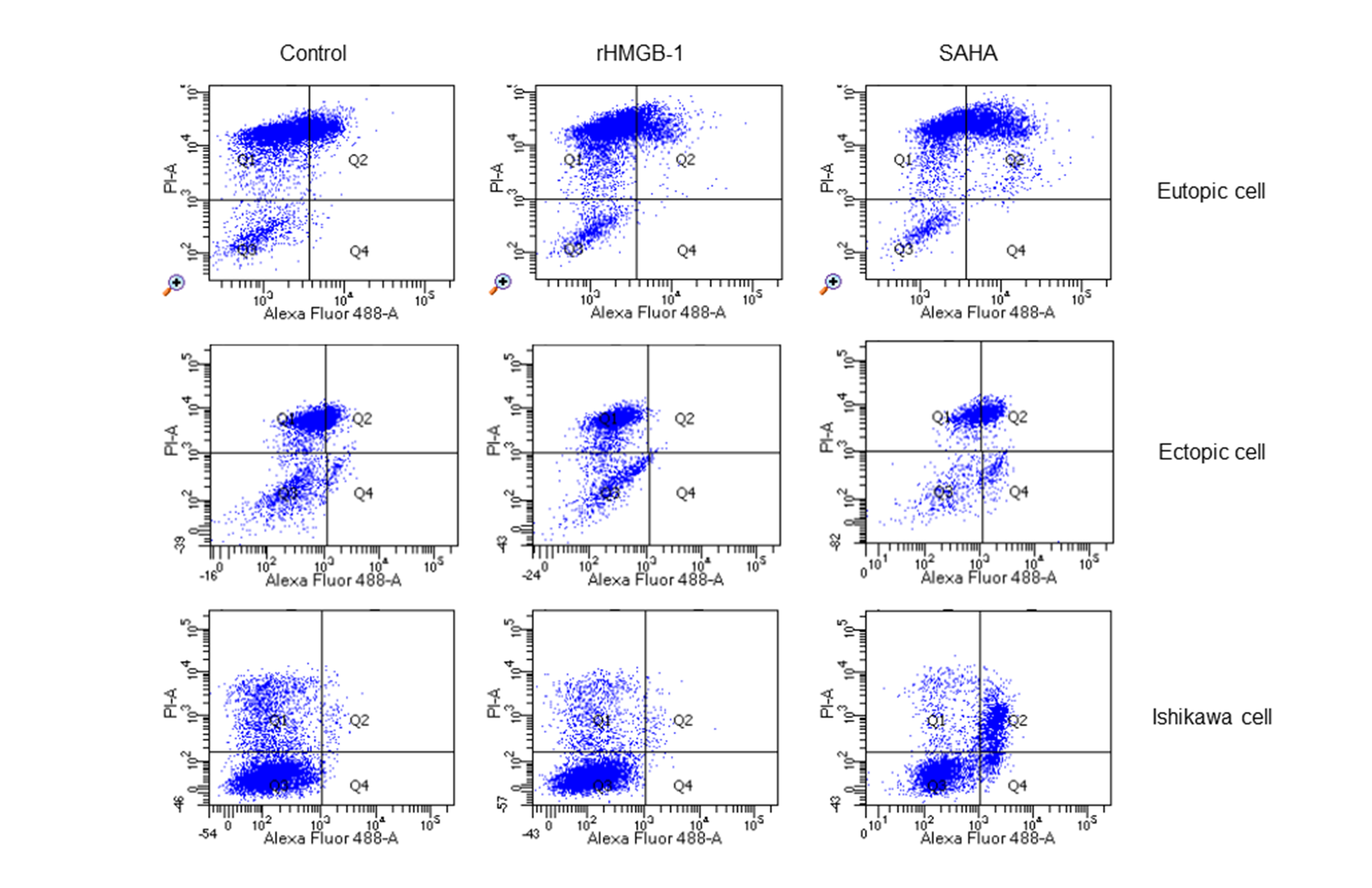

Supplement: Supplementary file 1 [file ijms-22-01427-s001.zip › Supple_fig1_0131.tif]

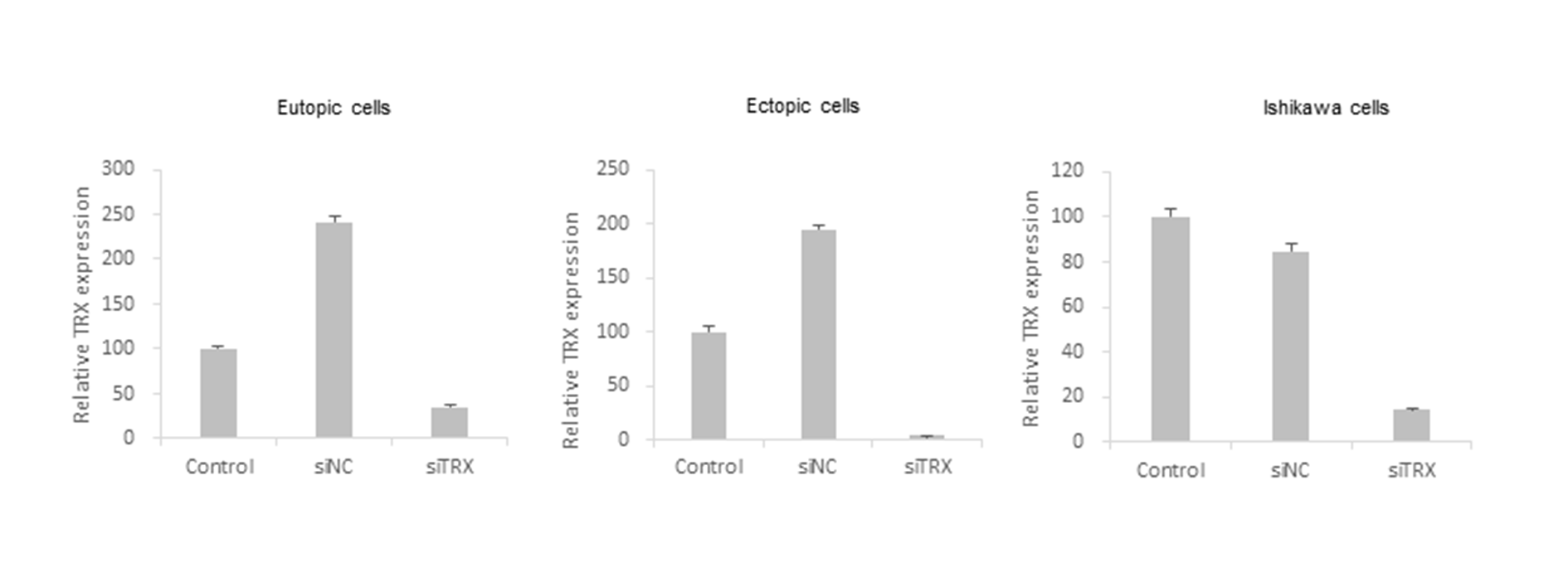

Supplement: Supplementary file 1 [file ijms-22-01427-s001.zip › Supple_fig2_0131.tif]

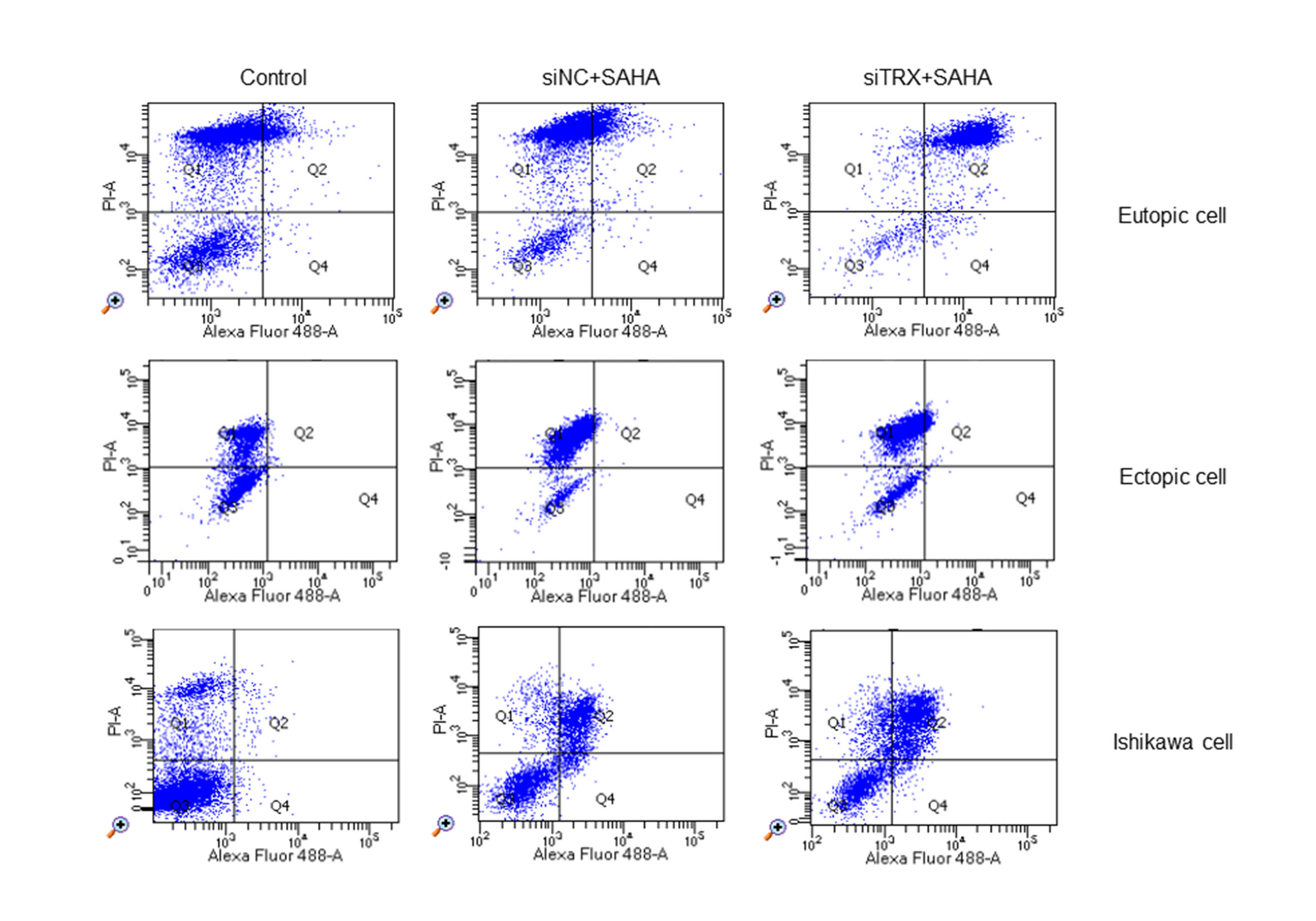

Supplement: Supplementary file 1 [file ijms-22-01427-s001.zip › Supple_fig3_0131.tif]
